# Smell and Taste in Severe CoViD-19: Self-Reported vs. Testing

**DOI:** 10.3389/fmed.2020.589409

**Published:** 2020-12-02

**Authors:** Andrea Mazzatenta, Giampiero Neri, Damiano D'Ardes, Carlo De Luca, Stefano Marinari, Ettore Porreca, Francesco Cipollone, Jacopo Vecchiet, Chiara Falcicchia, Vincenzo Panichi, Nicola Origlia, Camillo Di Giulio

**Affiliations:** ^1^Neurophysiology, Olfaction and Chemoreception Laboratory, Physiology and Physiopathology Section, Neuroscience, Imaging and Clinical Sciences Department, ‘G. d'Annunzio’ University of Chieti-Pescara, Chieti, Italy; ^2^Medicine and Aging Sciences Department, 'G. d'Annunzio'University of Chieti-Pescara, Chieti, Italy; ^3^Pneumology Unit, Ss. Annunziata Hospital, Chieti, Italy; ^4^Medical, Oral and Biotechnological Sciences, 'G. d'Annunzio'University of Chieti-Pescara, Chieti, Italy; ^5^Medicine and Aging Sciences Department, 'G. d'Annunzio'University of Chieti-Pescara, Chieti, Italy; ^6^Neuroscience Institute, National Council of Research, Pisa, Italy; ^7^Nephrology and Dialysis Unit, Unità Sanitaria Locale Toscana Nord Ovest-Versilia Hospital, Camaiore, Italy

**Keywords:** CoViD-19, smell, taste, smell test, olfactory threshold, hyposmia, anosmia

## Abstract

One of the most striking reported symptoms in CoViD-19 is loss of smell and taste. The frequency of these impairments and their specificity as a potential central nervous system function biomarker are of great interest as a diagnostic clue for CoViD-19 infection as opposed to other similar symptomatologic diseases and because of their implication in viral pathogenesis. Here severe CoViD-19 was investigated by comparing self-report vs. testing of smell and taste, thus the objective severity of olfactory impairment and their possible correlation with other symptoms. Because a significant discrepancy between smell and taste testing vs. self-report results (*p* < 0.001) emerges in our result, we performed a statistical analysis highlighting disagreement among normosmia (*p* < 0.05), hyposmia, severe hyposmia, and anosmia (*p* < 0.001) and, in hypogeusia and severe hypogeusia, while no differences are observed in normogeusia and ageusia. Therefore, we analyzed the olfactory threshold by an objective test revealing the distribution of hyposmic (34%), severe hyposmic (48%), and anosmic (13%) patients in severe CoViD-19. In severe CoViD-19 patients, taste is lost in 4.3% of normosmic individuals, 31.9% of hyposmic individuals, 46.8% of severe hyposmic individuals, and 17% of anosmic individuals. Moreover, 95% of 100 CoViD-19 patients objectively tested were affected by smell dysfunction, while 47% were affected by taste dysfunction. Furthermore, analysis by objective testing also highlighted that the severity of smell dysfunction in CoViD-19 subjects did not correlate with age and sex. In conclusion, we report by objective testing that the majority of CoViD-19 patients report severe anosmia, that most of the subjects have olfactory impairment rather than taste impairment, and, finally, that the olfactory impairment correlate with symptom onset and hospitalization (*p* < 0.05). Patients who exhibit severe olfactory impairment had been hospitalized for about a week from symptom onset; double time has taken place in subjects with normosmia. Our results may be limited by the relatively small number of study participants, but these suggest by objective testing that hyposmia, severe hyposmia, and anosmia may relate directly to infection severity and neurological damage. The smell test assessment could be a potential screening symptom that might contribute to the decision to test suspected cases or guide quarantine instructions, further therapeutic approach, and evaluation of neurological damage.

## Introduction

The World Health Organization (1) declared on January 30, 2020 a public health emergency caused by severe acute respiratory syndrome coronavirus type 2 (SARS-CoV-2), a β-coronavirus positive single-strand ribonucleic acid (+ss-RNA) of ~30 kilobases in length (2). This new human virus is the agent of coronavirus disease 2019 (CoViD-19) and, like other human coronaviruses, is responsible for 15% of all cases of acute viral nasopharyngitis. The common symptoms of the disease include fever, shortness of breath, smell and taste loss, cough, and fatigue; some patients develop severe pneumonia, acute respiratory distress syndrome, clot, and multiple organ failure resolving in death, though most affected individuals are healthy carriers without symptoms or with minor symptoms (3, 4). However, a plethora of information has been recorded: sociodemographic characteristics (ethnics, age, sex, lifestyle, e.g., smoking habits, etc.), pre-existing risk factors (heart, respiratory, and kidney failure, diabetes, neurological disease, cancer, etc.), and symptoms such as general (headache, cough, dyspnea, fever *T* > 38°C, nausea, vomiting, phlegm, chest pain, etc.), specific of an otolaryngologist (nasal obstruction, rhinorrhea, sore throat, etc.), and sensorial one, e.g., smell and taste essentially (5).

In about a week of symptom onset, approximately 70% of patients had recovered (3). Another characteristic of this novel infection is the distribution of receptor/receptor-associated enzymes that relate to the ability to spread R0, which estimates the number of people who can get infected from a single infected person: ~1.4/5.0 in SARS-CoV/S.-CoV-2, while ~1.347 in influenza and ~0.3–0.8 in MERS-CoV (6–9).

Initially, the target cells of SARS-CoV-2 have been suggested to be in the lower airway conversely to SARS-CoV (10). However, SARS-CoV and Middle East respiratory syndrome coronavirus (MERS-CoV) enters human host cells mainly *via* dipeptidyl peptidase 4, which is present in the lower respiratory tract, kidney, small intestine, liver, and cells of the immune system (7). Conversely, the SARS-CoV-2 protein characteristic spike binds to its cellular receptor, the angiotensin-converting enzyme 2 (ACE2), which is widely expressed in many cell types and organs like lung alveolar cells, nasal epithelium, cerebral cortex, digestive tract, kidney, gallbladder, testis, and adrenal gland (11). Viral entry occurs, as for other coronavirus, after the proteolytic cleavage of the spike (S) protein by the transmembrane protease TMPRSS2 (12, 13). Interestingly, ACE2 and TMPRSS2 show an extremely high expression in characteristic cells of the nasal epithelium, goblet, and ciliated cells. Accordingly, these cells are the candidates as loci of original viral infection and possible reservoirs for dissemination; in addition, SARS-CoV-2 is an enveloped virus that does not require cell lysis for viral release. Thus, the virus might exploit existing secretory pathways in nasal goblet cells for low-level, continuous release at the early stage with no overt pathology (14). In preliminary observations, obstruction of the olfactory cleft as a factor involved in increasing disease severity has been evaluated (5). A key element is understanding the pathophysiologic mechanisms underlying smell and taste impairment in CoViD-19, including potential viral spread through the olfactory neuroepithelium and invasion of the olfactory bulb and central nervous system (15).

In a previous similar infection, the MERS-CoV and SARS-CoV, neurological effect has been described and grouped in (i) direct effects per viral invasion of the central nervous system (CNS), (ii) secondary consequences due to failure of other organs that reflect on the CNS, and (iii) postinfectious and potentially immune-mediated complications (16–20). In CoViD-19, neurologic consequences occur, so far replicating the three outcome groups (21, 22).

Overall, the most striking reported neurological symptom in CoViD-19 is loss or weakening of smell and taste (22–24). Sudden dysfunctions in smell and taste have been described, first anecdotally, with marginal frequency smell in 5% and taste in 6% (21, 25), and then in retrospective reports which clearly indicated that the frequency was under-represented due to the evaluation system (23, 26, 27). In successive studies, smell and taste disorders are reported to be higher than 80% of infected patients and persist for about 7.1 ± 3.1 days (28, 29). The important discrepancy in these studies could be due to (i) the transient nature of the dysfunction, which could be due to the regenerative power of the olfactory sensory neurons (OSNs) and taste cells (30, 31), and (ii) the type and the time of testing from symptom onset. In household contacts of mildly symptomatic home-isolated SARS-CoV-2-positive patients, about two-thirds complain of an altered sense of smell or taste (32). A criticism is represented by the evaluation of the olfactory and gustatory function which has mainly been based on self-reported perception; further testing shows important procedural biases (33). Moreover, the objective evaluation of the sense of smell showed that self-reporting surveys may even underestimate the prevalence of anosmia in CoViD-19 patients (26, 27). Smell and taste alterations have led a consensus as an infection biomarker, even in the absence of other symptoms (34). Indeed higher viral loads have been detected in nasal swabs, compared to those obtained from the throat, in both symptomatic and asymptomatic patients. Consequently, the nasal epithelium is considered as a crucial site for initial infection and may serve as a key reservoir for viral spread across the respiratory mucosa and for viral transmission (35). In transgenic mice expressing the human SARS virus receptor (ACE2) and infected with SARS-CoV, neuro-invasion from olfactory to cardiorespiratory centers in the medulla has been showed (36).

The objective determination of chemoreceptive impairments is an important tool for CoViD-19 infection diagnosis for its specificity as a potential CNS virosis and for understanding the pathogenesis (27). In the present study, severe CoViD-19 was investigated by comparing self-report vs. testing of smell and taste with their possible correlation with age, sex, and other symptoms.

## Materials and Methods

The controlled cross-sectional study approved by the local Ethics Committee (n. richtr25p), performed according to Italian laws, follow the Helsinki Declaration (2013).

In the study, the smell and taste of 100 patients (mean age, 63 ± 15 SD; range 28–94 years old; 70 males and 30 females) with severe CoViD-19 infection and in assisted breathing, oxygenotherapy, at “Ss. Annunziata” University Hospital CoViD-19 special ward were evaluated. The infection was confirmed by SARS-CoV-2 RNA by RT-qPCR laboratory testing (days from positive swab range 5–10). The patients were defined as a case of severe CoViD-19 if they have clinical signs of severe pneumonia: fever, cough, dyspnea, and fast breathing plus one of the following symptoms—respiratory rate >30 breaths/min, severe respiratory distress, or SpO_2_ < 90% on room air (37, 38) and requiring oxygenotherapy and blood pressure monitoring in internal medicine (or intensive care units, not our case) (39); severe CoViD-19 patients are about 15% of the total infected cases (1).

The contemporary not hospitalized control groups, CoViD-19 negative, selected *ad hoc* for this study are positive controls (*N* = 16; mean age, 64 ± 5.8 SD, range 49–70 years old; 12 males and four females), subjects with renal failure (in dialysis) who characteristically suffer from smell impairment and taste disturbances (39–41), and negative controls (*N* = 50; mean age 28 ± 2 SD; range 24–32 years old; 31 males and 29 females) who are healthy subjects recruited among medicine and nursing students (Table 1). In order to avoid the biases represented by (i) comorbidity and neuro-functional decrement in aged subjects and (ii) olfactory phenotype variation within age, we uses a young population as negative control because a high prevalence of olfactory threshold normosmia is expected (42).

**Table 1:** Clinical data from CoViD-19 patients, positive control for smell impairments (the diabetes group), and negative control (the healthy subjects).

	**CoViD-19** ***N* = 100** ** (70 males, 30 females)**	**Positive control** ***N* = 16** ** (12 males, 4 females)**	**Negative control** ***N* = 50** ** (31 males, 29 females)**
**Sociodemographics**			
**Nationality**	Italian	Italian	Italian
Mean age ± SD	63 ± 15	64 ± 5.8	28 ± 2
Age range (years)	28–94	49–70	24–32
**Pre-existing risk factors**			
Active smoker (%)	20	2	18
**Metabolic disease**			
- Hypertension and chronic heart failure	25	12	–
- Respiratory disease^a^	19	8	7
- Diabetes	15	6	–
- Obesity	12	5	4
- Chronic kidney disease	5	16	–
- Cancer	3	–	–
- Rheumatic disease	1	–	–
- Other	1	1	–
Neurological disease^b^	8	1	–
**Vital signs (mean** **±** **SE)**			
Heart rate	82.65 ± 0.92	73.4 ± 2.35	62 ± 1.05
Blood pressure, minimum	72.94 ± 0.75	70.46 ± 4.13	70.4 ± 1.3
Blood pressure, maximum	120.37 ± 1.39	131.4 ± 7.0	110.4 ± 0.94
Respiratory rate (breaths/min)	18.19 ± 0.28	14 ± 0.41	12 ± 0.5
Temperature (°C)	>38°C	<38°C	<38°C
**Laboratory results on ambient air (mean** **±** **SE)**			
pO_2_ (mm Hg)	67.98 ± 1.3	87.2 ± 5.1	96.61 ± 4.3
pCO_2_ (mm Hg)	39.39 ± 1.04	37.36 ± 3.2	35.21 ± 4.7
Hb	12.53 ± 0.19	11.8 ± 0.07	15.3 ± 1.46
Lactate (mmol/l)	1.4 ± 0.2	1.0 ± 0.5	0.8 ± 0.32

a *Asthma, chronic obstructive pulmonary disease, obstructive sleep apnea, etc*.

b *Dementia, Parkinson's disease, Alzheimer's disease, visive disturbances, etc*.

Following informed consent, the patients were enrolled in the study at hospitalization. A self-report smell and taste perception was assessed by a simple questionnaire [question: Do you smell (or taste)? Answer: no or yes], while olfactory and gustatory tests were performed by using a disposable four-item Olfactory Smart Threshold (O.S.T. test Asteria Healthcare) based on the Connecticut Chemosensory Clinical Research Center (C.C.C.R.C.) threshold test (43) and the Italian population age phenotype threshold test (42) and a disposable homemade two-item suprathreshold taste test (0.5 g/ml sucrose and 0.5 g/ml sodium chloride). The taste test ratio follows that ACE2 inhibitors can induce ageusia with a complex mechanism which involves G-protein-coupled protein and sodium channels present in the taste buds (44). The OST test consists in a logarithmic scale of n-butanol to assess by positive answer at a green vial that is considered as normosmia, yellow for hyposmia, and red as the severe hyposmia threshold; no answer means anosmia. The white odorless vial is the test's negative control (see additional material; OST test, the App with the guide, and an example are shown). The taste test consists in a taste adsorbed band of 200 ul solution placed at the middle of the anterior tongue. The participant is to report the smell or taste perceived as yes or no by pointing at the corresponding word on a response chart. The negative control group was used for test–retest reliability, within 15 days, which is OST test = 0.89 and taste test = 0.99 Pearson correlation.

Furthermore, the following parameters and symptoms were recorded: age, sex, days to symptom onset, headache, nausea, vomiting, breathing difficulties, and rhinorrhea as summarized by Lovato and de Filippis (4). The results of testing were correlated to the clinical parameters and the information obtained by a medical interview, i.e., onset of symptoms recorded at hospitalization. A trained operator at “Ss. Annunziata” University Hospital CoViD-19 special ward administered the 5-min experimental session per subject, which included self-report and objective tests; the patients were left free to stop the testing.

Statistical elaboration, test–retest reliability Pearson correlation, MANOVA, and one-way ANOVA were used, with the α-level set to 0.05; *p* < 0.05 was regarded as significant. Commercial software statistical packages were used for all the data and statistical analyses (IBM SPSS, NY, USA; OriginLab Co., Northampton, MA, USA).

## Results

### Quantitative Testing vs. Self-Reporting

**Figure 1:**
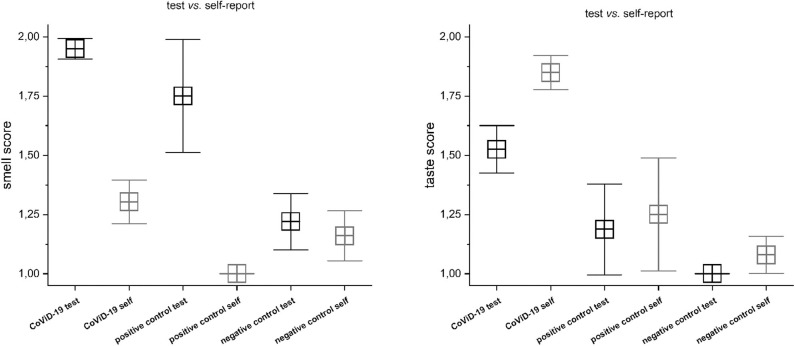
Comparison between testing vs. self-report in CoViD-19, positive control, and negative control in smell **(Right)** and taste **(Left)**. Significant discrepancy between smell testing vs. self-report, MANOVA *p* < 0.001 (for detailed statistic, see text).

The CoViD-19 patients show a significant discrepancy between smell testing vs. self-report, MANOVA *p* < 0.001, *F*_(1, 198)_ = 158.03 (mean quantitative testing 1.05 ± 0.22 SD; mean self-report 1.70 ± 0.46 SD) (Figure 1). A series of *post hoc* one-way ANOVA returned a significant disagreement among all groups: quantitative testing (q.t.) vs. self-reporting (s-r) normosmia [*F*_(1, 9)_ = 6.0] *p* < 0.05; q.t. vs. s-r hyposmia [*F*_(1, 69)_ = 91.67], q.t. vs. s-r severe hyposmia [*F*_(1, 95)_ = 103.4], and q.t. vs. s-r anosmia [*F*_(1, 23)_ = 33.0], *p* < 0.001. The positive and the negative controls show a significant discrepancy between smell testing vs. self-report: *p* < 0.05 in normosmia and hyposmia and *p* < 0.001 only in the negative one.

A significant disagreement was also assessed between taste testing vs. self-report: MANOVA *p* < 0.001, *F*_(1, 198)_ = 33.7 (mean testing 1.49 ± 0.49 SD; mean self-report 1.85 ± 0.36 SD) (Figure 1). A series of *post hoc* one-way ANOVA returned no differences among normogeusia [*F*_(1, 9)_ = 1.0], *p* =0.35, and ageusia [*F*_(1, 23)_ =0.19], *p* =0.67, while significant differences were observed in hypogeusia [*F*_(1, 69)_ = 28.72], severe hypogeusia [*F*_(1, 95)_ = 12.68], *p* < 0.001. The positive control does not show significant differences, while the negative control has significant incongruity in hyposmia (*p* < 0.05).

Consequently, the following analysis was performed on objective testing results.

### Smell and Taste Dysfunction

**Figure 2:**
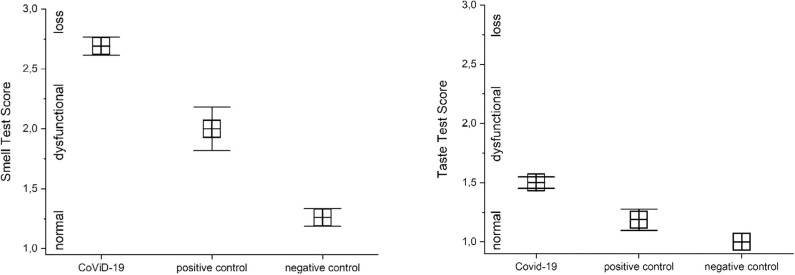
Comparison of quantitative smell and taste test results in severe CoViD-19 patients and positive and negative controls. Significant differences occur between severe CoViD-19 patients and controls, MANOVA *p* < 0.001 (for detailed statistic, see text).

A preliminary analysis of variance returned a significant correlation between CoViD-19 infection and smell and taste disfunctions, *p* < 0.001 [*F*_(1, 161)_ = 47.2 and *F*_(1, 161)_ = 17.2]. A detailed analysis in CoViD-19 patients revealed that 95% show smell dysfunction, 75% in positive control and 22% in negative control (analysis on olfactory threshold is in Olfactory Threshold). There were significant differences in smell between CoViD-19, positive control patients, and negative control subjects: MANOVA *p* < 0.001, *F*_(2, 165)_ = 70.9. A series of *post hoc* ANOVAs returned significant differences between CoViD-19 and positive control patients, *p* < 0.001, *F*_(1, 115)_ = 11.5 (mean CoViD-19 2.69 ± 0.76 SD, mean positive control 2.0 ± 0.73 SD); CoViD-19 patients and negative control, *p* < 0.001, *F*_(1, 149)_ = 142.1 (mean negative control 1.26 ± 0.53 SD); and positive control patients and negative control, *p* < 0.001, *F*_(1, 65)_ = 19.7 (Figure 2).

In CoViD-19 patients, 47% showed taste dysfunction, while it was 12.5% in positive control and none in negative control. There were significant differences in taste between CoViD-19, positive control patients, and negative control subjects, MANOVA *p* < 0.001, *F*_(2, 165)_ = 27.7. A series of *post hoc* ANOVAs returned significant differences between CoViD-19 and positive control patients, *p* < 0.05, *F*_(1, 115)_ = 6.04 (mean CoViD-19 1.50 ± 0.49 SD, mean positive control 1.89 ± 0.36 SD); CoViD-19 patients and negative control, *p* < 0.05, *F*_(1, 149)_ = 52.5 (mean negative control 1.0 without SD); and positive control patients and negative control, *p* < 0.05, *F*_(1, 65)_ = 14.1 (Figure 2).

**Figure 3:**
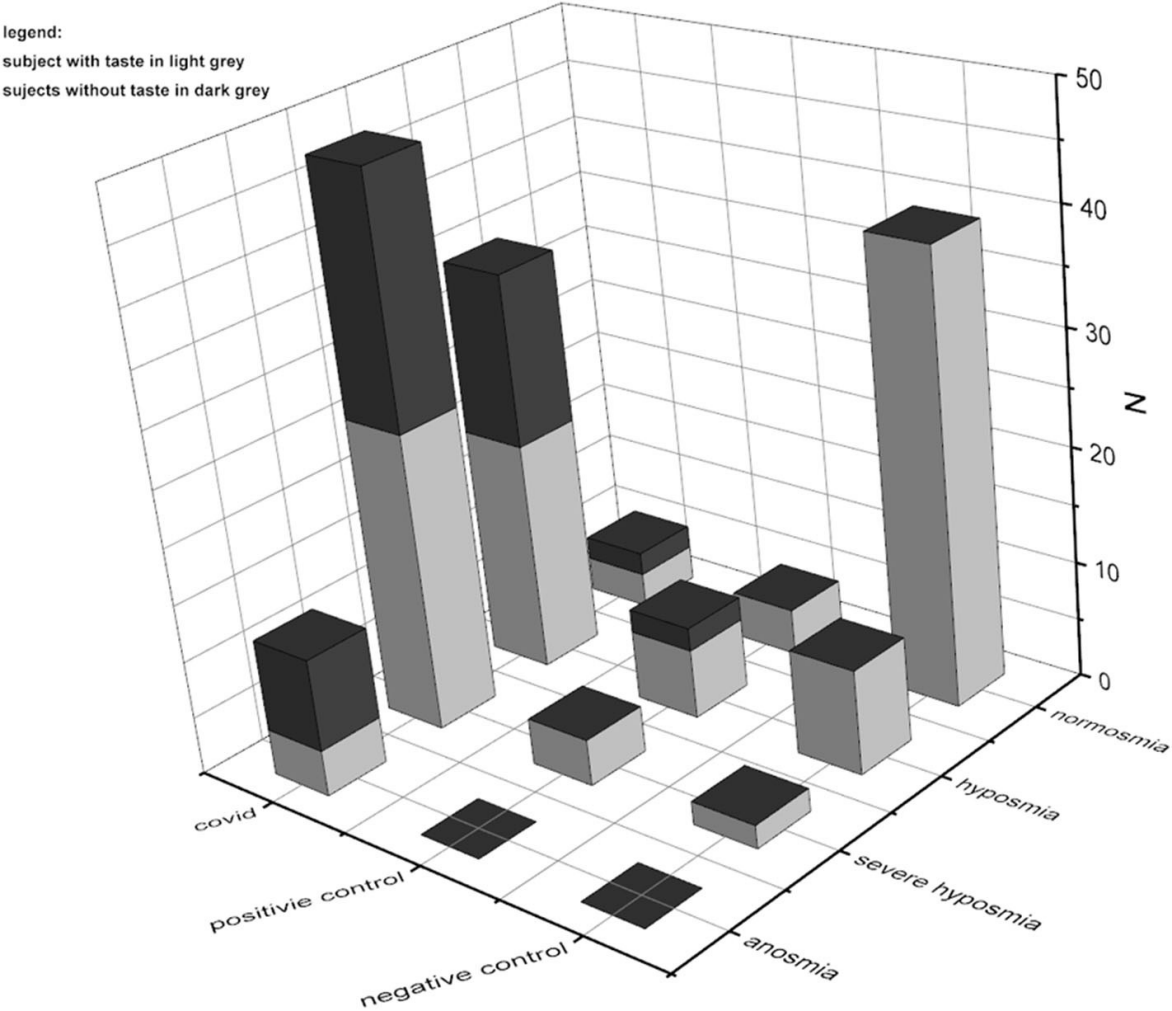
Comparison of quantitative smell and taste test results in severe CoViD-19 patients and positive and negative controls. The prevalence of smell impairments is clear; besides in CoViD-19, taste disturbances are present also in normosmia.

In normosmia, severe CoViD-19 patients' taste is preserved in 5.7%, while it is lost in 4.3%; in hyposmia, severe CoViD-19 patients' taste is present in 37.7% and is lost in 31.9%; in severe hyposmia, severe CoViD-19 patients' taste perception was still in 49.1%, while it was not in 46.8%; in anosmia, severe CoViD-19 patients' taste persists in 7.5%, while it is lost in 17%. Comparing with controls, there was a significant difference for taste perception in normoxia only between CoViD-19 and negative control, *p* < 0.001, *F*_(1, 43)_ = 46.5; in hyposmia, between CoViD-19 and negative control, *p* < 0.05, *F*_(1, 43)_ = 7.7, and between positive and negative controls, *p* < 0.05, *F*_(1, 16)_ = 6.5; in severe hyposmia, no differences emerged; and finally, no comparison could be assessed in anosmia because in controls there was no taste loss (Figure 3).

### Olfactory Threshold

**Figure 4:**
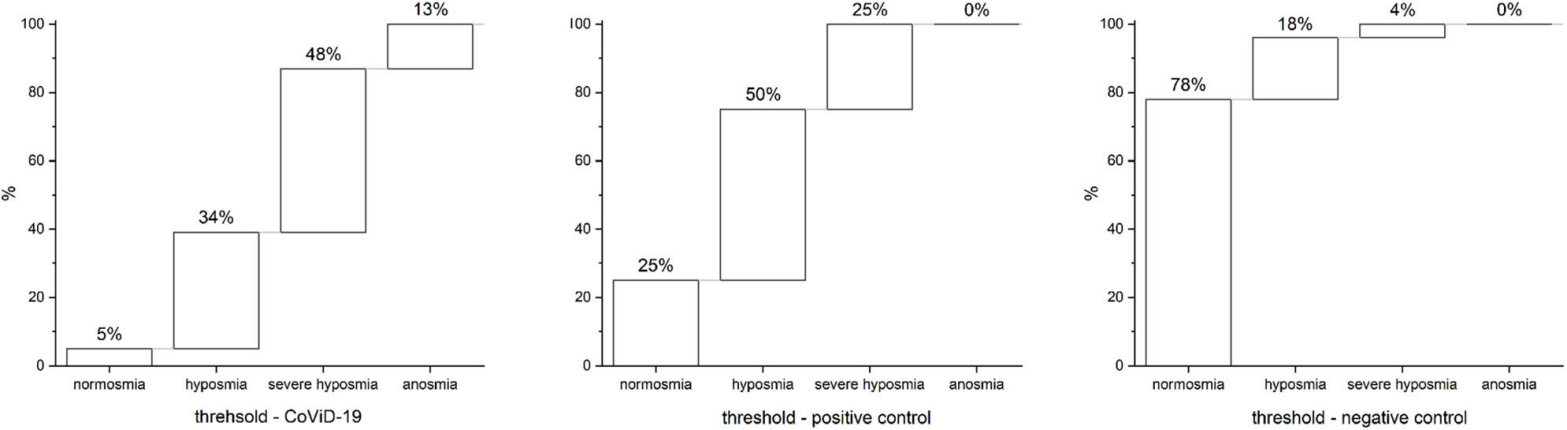
Olfactory threshold in severe CoViD-19 patients, positive and negative controls. A total of 95% of severe CoViD-19 patients show olfactory impairment in comparison to 75% of positive control and 22% of negative control. A detailed percentage for olfactory threshold is shown for CoViD-19 patients and positive and negative controls. Highlight of the prevalence of severe hyposmia in CoViD-19 patients, which is a characteristic compared to other smell-dysfunctional patients like the positive control that has a prevalence in hyposmia. Hyposmia and sever hyposmia are slightly present in negative controls—healthy subjects.

In 95% of assisted-breathing patients with severe CoViD-19 infection, smell impairments occur; 34% were identified as hyposmic, 48% as severe hyposmic, and 13% as anosmic, with a prevalence of severe hyposmia. In the positive control, 75% had smell disturbances with prevalence of hyposmia in 50% and severe hyposmia in 25%, while no anosmia was reported. In negative controls, only 22% reported smell alteration: 18% were hyposmic and 4% were severely hyposmic; no anosmia was found (Figure 4). Statistical analysis returns significant differences (*p* < 0.05) within and between groups.

#### Threshold vs. Age

**Figure 5:**
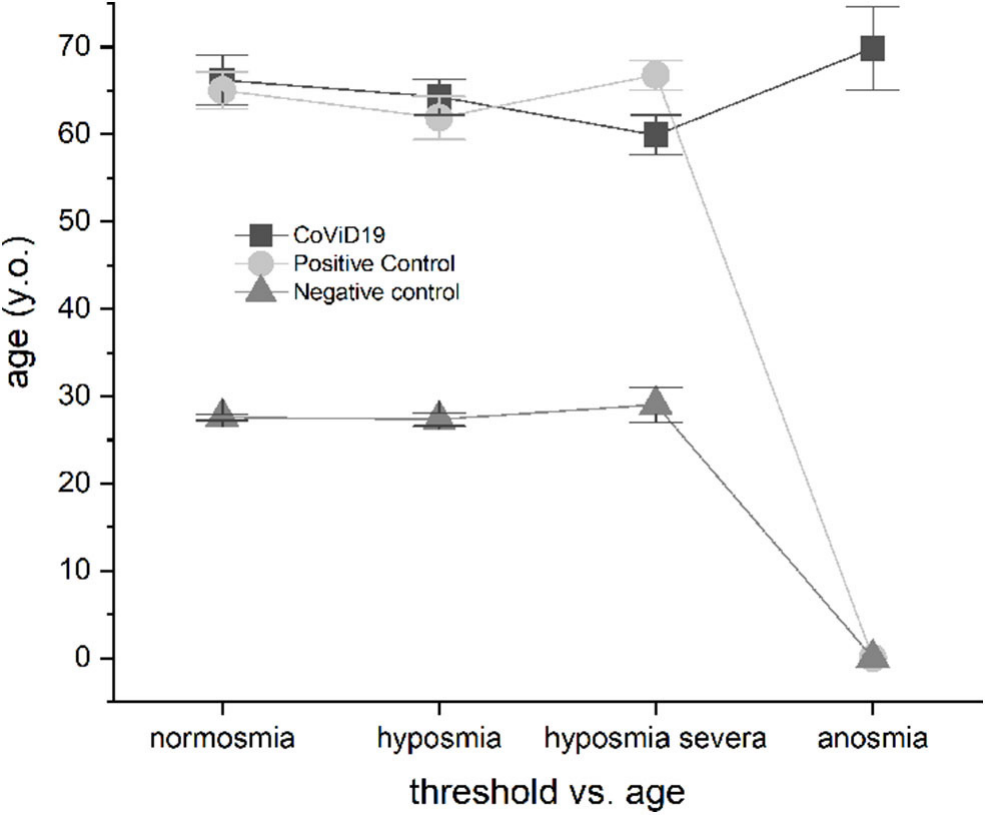
Olfactory threshold vs. age in severe CoViD-19 patients and positive and negative controls; no age correlation emerged.

The CoViD-19 patients and positive control did not show differences in normosmia, hyposmia, severe hyposmia, and anosmia related to age, while both showed significant differences with the negative control (*p* < 0.001) (Figure 5).

#### Smell Threshold Impairment vs. Sex

**Figure 6:**
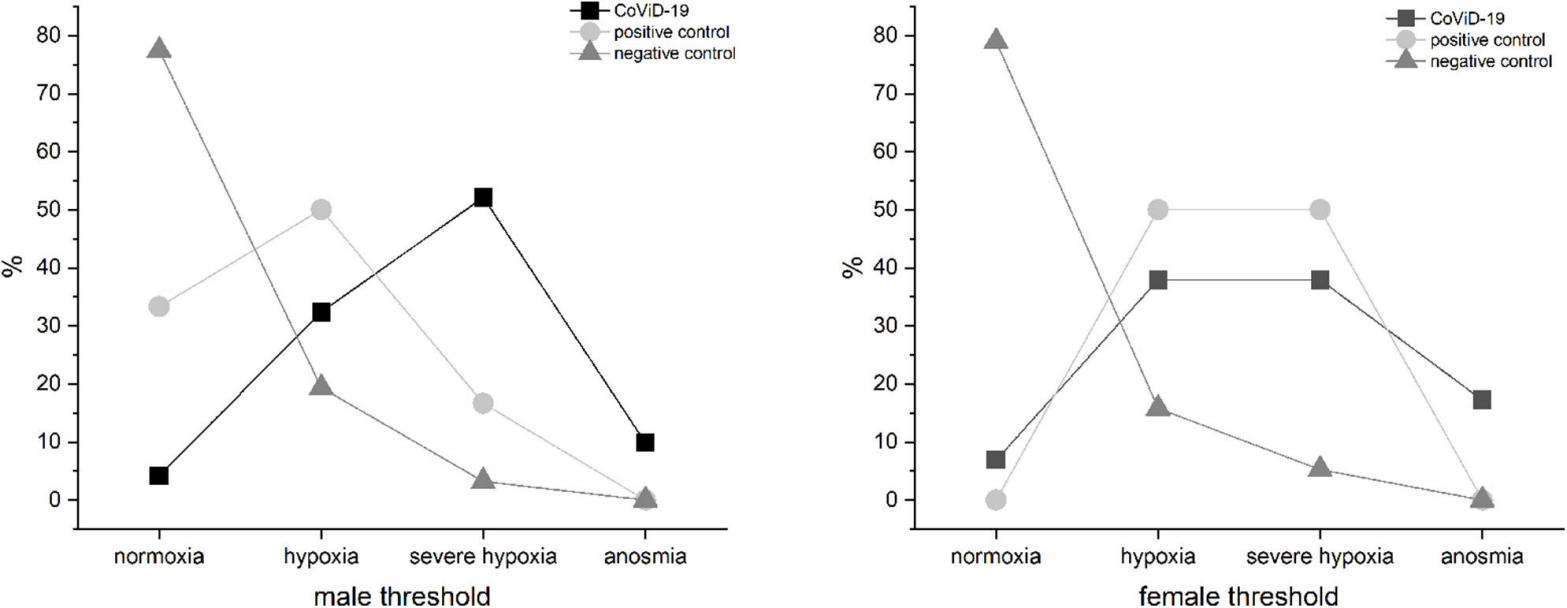
Olfactory threshold vs. sex in severe CoViD-19 patients and positive and negative controls; no sex correlation emerged.

The CoViD-19 patients, positive control, and negative control did not show differences related to sex (Figure 6); the only discriminant is infection.

#### Smell Threshold Impairment vs. Symptom Onset to Hospitalization

**Figure 7:**
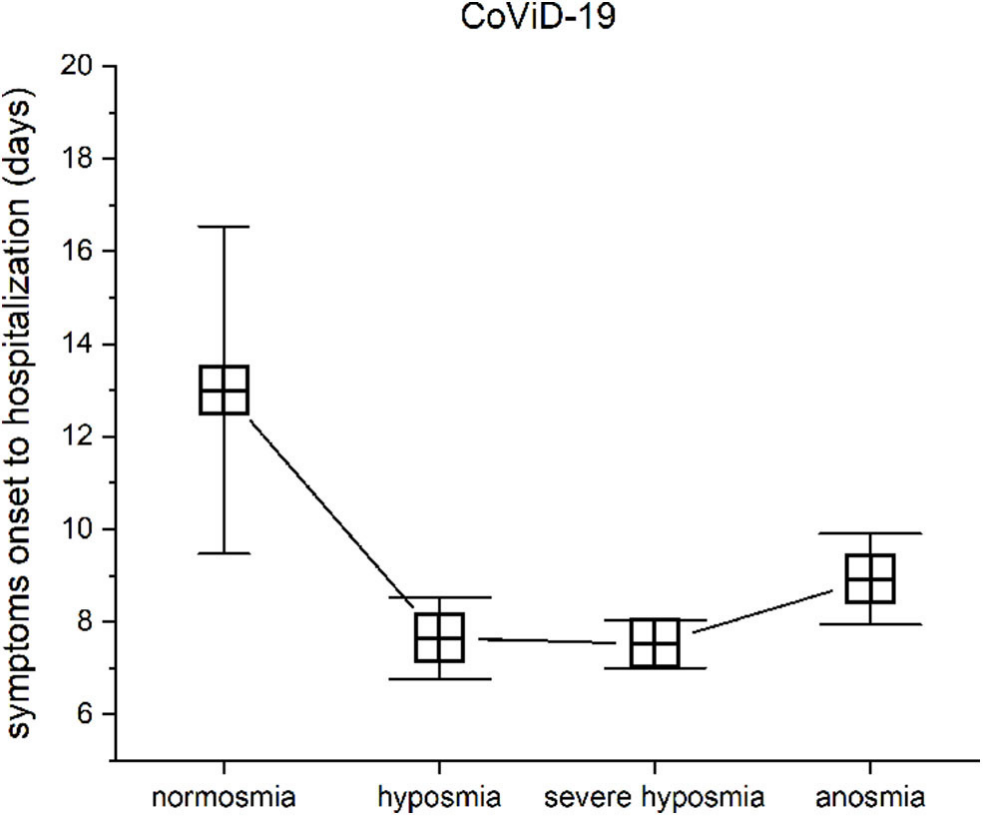
Olfactory threshold *vs*. symptom onset to hospitalization in severe CoViD-19 patients. The days from symptom onset to hospitalization between patients with normosmia vs. threshold impairment are statistical significant.

Patients with mild-severe olfactory impairment had been hospitalized in about a week from symptom onset: hyposmic patients show a mean of 7.65 ± 5.18 SD, severe hyposmic patients had 7.52 ± 3.59 SD, and anosmic patients had 8.92 ± 3.4 SD, while in normosmic severe CoViD-19 patients, symptom onset to hospitalization approximately occur about 13 days ± 3.54 SD. These differences in days from symptom onset to hospitalization were found significant with the level of the olfactory impairments, MANOVA *p* < 0.05, *F*_(3, 99)_ = 2.86. In particular, a *post hoc* one-way ANOVA series returned significant differences in normosmia vs. hyposmia [*p* < 0.05, *F*_(1, 38)_ = 4.94], normosmia vs. severe hyposmia [*p* < 0.05, *F*_(1, 52)_ = 10.6], and normosmia vs. anosmia [*p* < 0.05, *F*_(1, 16)_ = 4.98], while no differences in hyposmia vs. severe hyposmia and anosmia and in severe hyposmia vs. anosmia was found (Figure 7).

## Discussion

Smell and taste failures have been indicated as highly prevalent and distinctive symptoms of CoViD-19 (23, 24, 33). Therefore, a growing interest in the use of these sensorial impairments to investigate SARS-CoV-2 infection had led to a wide consensus (27, 34). A plethora of research, mostly anamnestic, about the significant prevalence of smell and taste alteration in CoViD-19 patients higher than in other viral infections or controls have been published. These studies range from large epidemiological study, observational case series, case-controlled and cross-sectional studies to electronic surveys, with significant methodological and result differences, e.g., smell loss range from 5.1 to 85.6% [for these studies' analysis, see (45)]. Conversely, chemosensory investigation is critical to establish the presence and the degree of dysfunction, the veracity and accuracy of a patient's self-report, the efficacy of treatments, and the degree of functional recovery. The lack of objective quantification of smell and taste led many countries to not include chemosensory disorders in the CoViD-19 diagnostic guidelines (34). The principle of such investigation should be screening individuals with objective quantification by a reproducible disposable system that is easy to use, fast, and cheap. This will offer the opportunity to correlate test results with biometric parameters, symptoms, and other physiological signs and also correlate smell and/or taste variation with severity of disease. Considering the level of danger associated with SARS-CoV-2, a reliable, disposable, and fast test to discriminate smell vs. taste impairments is required; accordingly, we settle with two quantitative, fast, and disposable tests that we compare with a self-report questionnaire. In this perspective, we will discuss our finding in comparing quantitative testing vs. self-reporting, smell and taste evaluation, and threshold smell test and its correlation with sex, age, symptoms onset. Overall, we found a significant impairment of olfactory and gustatory functions in CoViD-19 compared with the positive control, which will be accurately analyzed in the following sections.

### Quantitative Testing vs. Self-Reporting

The landmark works demonstrate fallacy in self-evaluation of smell and taste because most individuals are inaccurate in assessing the nature and the degree of their chemosensory problem (46, 47). For instance, olfactory and taste cross-modal perception is crucially biased, as well as olfactory-trigeminal and taste-somatosensorial sensitivities as other odds; in addition, the molecular properties, e.g., concentration and volatility, highlight a complicated, multidimensional picture (48). Nonetheless, self-reporting is still used, while quantitative testing is rare (49). Even so, clinical tests of olfactory and gustatory systems are available and allow one to quantitatively measure the degree of sensory impairment (48, 49). We compared olfactory and gustatory self-reporting and quantitative testing in the same groups of CoViD-19 patients, positive controls, and negative controls in order to verify the possible convergence between these tests. Our results show a significant disagreement in both smell and taste assessments. Importantly, failure is observed in anosmia, severe hyposmia, hyposmia, and normosmia self-evaluation. These results are in full agreement with previous works (46, 47). We found it scarcely informative and therefore unsafe to use self-evaluation in CoViD-19 as it is a hazardous disease. Accordingly, without quantitative testing, the accuracy of a patient's chemosensory complaint cannot be definitively established; the efficacy of therapeutic interventions cannot be correctly established as well. Furthermore, as abundantly demonstrated by quantitative testing, smell impairments are generally more common than taste impairments. In fact, most patients who complain clinically of a “taste” disturbance have altered smell function [e.g., (50, 51)] because the flavor of foods largely depends upon volatiles that reach the olfactory receptors *via* the nasal pharynx during mastication and deglutition, which is wrongly interpreted as taste (46, 47). It is necessary therefore to perform a clinical assessment of patients who have chemoreceptive complaints by quantitative tests in order to discriminate between smell and taste disorders and determine the severity of alteration.

Consequently, following is a discussion of the results on the objective testing performed.

### Smell vs. Taste Dysfunction

The distinction between solely olfactory loss, the inability to perceive volatile odorants, and the true gustatory loss of bitter, sweet, salty, sour, umami, and fatty acid long chain sensitivity or their decrement is a critical issue. Unfortunately, this division is often underappreciated; usually, a clinical evaluation of a patient who complains of taste loss or its decrement is usually dependent on olfactory dysfunction (52, 53). Smell disorders are more frequent because of the vulnerability and the anatomical distinctiveness of the olfactory system as well as the physiological decline during aging (42, 54). Several quantitative tests are available for olfactory (e.g., C.C.C.R.C., U.P.S.I.T.) and gustatory testing (e.g., spatial tests, taste sticks, tasting tablets) (53). In this study, we have used quantitative, disposable, fast, and easy tests on an Italian population (42) that allows to discriminate between smell and taste perceptions. In assisted-breathing patients with severe CoViD-19 infection, we found that the prevalence of olfactory dysfunction was about two times than gustatory disturbances. Our results for the positive controls agree with a previous study showing the important reduction of the olfactory capacity in patients who are on hemodialysis (40). The prevalence of salt taste dysfunction is about 58% in hemodialysis patients (41), which agrees with our results. Furthermore, we agree with a large cohort study, the Epidemiology of Hearing Loss Study (EHLS), including olfactory testing, that found 24.5% olfactory impairment in a healthy population (55) and with studies on taste [for a review, see (52)]. However, our findings for taste are not in agreement with the prevalence reported by Lechien et al. (29) that was higher compared to our data. This could be due to the use of a questionnaire which underestimated olfactory impairments and overestimated the gustatory ones with respect to a quantitative test. Vaira et al. (26), in a multicentric study, report a similar result on smell impairment of about 70% and taste of about 45% by using a patient's homemade test. Interestingly, another debatable aspect is the combined smell and taste disorders that are significant in infection by SARS-CoV-2. We have analyzed the gustatory disturbances in correlation with olfactory impairments, and our results agree with a previous study indicating that the infection preferentially affects smell (27, 33, 34). Taste disturbance increases with the severity of smell alterations, which agrees with the different methodological study of Vaira et al. (26). Our results demonstrate that a high proportion of CoViD-19 patients describe a taste disturbance; however, in contrast with a previous study, standardized functional testing of the gustatory modalities of sweet and salty did not reveal a gustatory loss. On the contrary, the subjectively altered taste is most likely caused by impaired retro-nasal olfaction. This interpretation agrees with Hintschich et al. (56), suggesting that CoViD-19 is closely associated with olfactory damage rather than with gustatory loss when tested psychophysically. Taste-responsive neurons in the nucleus of the solitary tract (NTS) also respond to odorants in an odorant-specific manner (57). The influence of olfactory stimuli on taste responses in the NTS provides evidence of a widespread modulation of odorants on taste response magnitude and latency when tastants are presented in tandem with odorants (58). Consequently, taste is driven, reinforced, and modulated by cross-modal olfaction perception, and its impairment could reflect smell injury (59). Future studies should be addressed to investigate a correlation between the severity of smell impairment and its correlation with taste disturbance.

### Olfactory Threshold

The evaluation of patients with smell complaints is difficult without standardized quantitative methods of assessment. Smell can be evaluated by butanol ascendant concentration as characteristic of most psychophysical tests used (60). Overall, studies on test–retest reliability in nine quantitative smell tests show, in healthy subjects, that nominally distinct tests of olfactory function are measuring a common source of variance (61). Thus, given the olfactory complexity, we use a highly reliable quantitative smell test that is disposable, fast, and easy to use, based on butanol ascendant concentration, allowing to discriminate smell variation: hyposmia, severe hyposmia, and anosmia. In agreement with other studies, we found a lower rate of anosmia in comparison with severe hyposmia and hyposmia (39), probably related to the regenerative nature of OSNs and/or the severity of infection and/or individual immunological resistance. It has been hypothesized that a disease dichotomic behavior correlated with SARS-CoV-2 serum IgG (39) in immune responses to the virus at the level of the nasal and olfactory mucosa. Nasal immune responses could block viral replication and dissemination to the lower respiratory tract, producing a local inflammation involving the olfactory epithelium region, while on the contrary, if viral replication spreads to the lower respiratory tract, this leads to a systemic immune response and inflammation (39). Furthermore, CoViD-19 patients with a similar clinical condition show different olfactory thresholds because of different immune responses or it could be related to viral penetration in CNS as suggested by Mao et al. (21).

In assisted-breathing patients with severe CoViD-19, we found 95% of smell impairments, in agreement with Moein et al. (27) who reported 98%. Furthermore, to the best of our knowledge, this controlled study shows for the first time the relative abundance of hyposmia, severe hyposmia, and anosmia in severe CoViD-19 hospitalized patients by using a quantitative test.

#### Threshold vs. Age

In mild-to-moderate COVID-19, it has been reported that symptoms vary according to the age and the sex characteristics of patients (39).

In a previous large study, we identified olfactory threshold phenotypes: young, mature, and elder, pointing out that smell in aging has non-linear variations and non-normal distribution (42). A possible cross-effect between olfactory phenotype and CoViD-19 is excluded because most of the patients are aged. Accordingly, we did not find any effect of age, in agreement with another study (62).

#### Threshold vs. Sex

Sex differences in smell ability are reported, although not due to ethnic or cultural factors *per se* (63). To ascertain the likelihood of a sex difference in the smell ability of CoViD-19 patients, the test results were analyzed, and no sexual variance emerged. The only discriminant seems to be infection, which is in agreement with another study (26).

#### Threshold vs. Symptom Onset

In common with most medical disorders, it is important to determine, as best as possible, the time of onset of the symptom, its severity, and the comorbidities to understand the underlying pathophysiology. The clinical onset of chemosensory disorders occurs characteristically in the very early stages of infection, generally within the first few days (21, 26). Most of research focus on this aspect and to find a correlation between nasal obstruction or rhinitis symptoms in mild or moderate cases (23, 24, 27, 29, 62). Conversely, in hospitalized patients, shortly from onset symptoms we found an important decrement in olfactory threshold, while patients with normosmia have had a longer period before hospitalization from symptom onset. The interpretation of this outcome could be due to viral charge or to a general individual resistance. However, speculating on the aspect of a high viral charge, smell fallacies could be crucial as predictive of a rapid severe infection. In contrast with the current idea that chemosensory symptoms were the first sign of an infection [e.g., Vaira et al. (62)], we found normosmia in severe hospitalized patients. It is possible that olfactory impairment was recovered at the moment of testing because of the longer time before the hospitalization of these subjects. In fact, chemosensitivity recovery occurred in <7 days in 74.6% of cases (e.g., 26, 59). Normosmic severe patients had been hospitalized in about 2 weeks since symptom onset, while other patients had been hospitalized in about a week. Stressing this aspect, we should remark the fact that testing was performed at hospitalization, a sort of point zero. All clinical and physiological information were acquired at this time and recorded for future study of the pathology evolution. In particular, in acute cases characterized by a fast rise of severe respiratory distress, fever, cough, dyspnea, and fast breathing, the olfactory disturbances are clearly evaluable by objective testing, while it is not so in “slower” cases characterized by a longer time from symptom onset to hospitalization; probably, smell was already recovered. Future studies should address this important issue with objective quantitative tests.

In conclusion, these results demonstrate that a high proportion of CoViD-19 patients have olfactory impairment, in agreement with most of the published papers, further describing a taste disturbance that correlates with smell impairment severity. However, in contrast with prior publications, standardized functional testing of the smell and taste modalities shows that most of gustatory loss is caused by impaired retro-nasal olfaction. The discrepancy among studies could be due either to the testing systems (here we clearly suggest an objective one) or the timing of investigation from symptom onset because of the regenerative power of the OSNs and taste cells (30, 31). Our results may be limited by general aspects: the relatively small number of study participants, the single institution that however received patients from all over the region, the homogeneous patient population of Italians with age ranging around 60 years old. However, the *pandemia per se* suggests that race, age, and country do not limit virus dissemination. Furthermore, this work focuses only on severe CoViD-19 patients because of lack of information comparing other so-called asymptomatic/pre-symptomatic and mild/moderate patients. The outcome of this study could be interesting for a re-evaluation of other disease conditions in view of early detection of potential severe patients. Consequently, further studies should compare severe to other disease conditions in a cross-sectional multicenter study and embrace other physiological parameters in correlation with olfactory impairment.

Nevertheless, our findings agree with a recent study on hospitalized CoViD-19 patients (27). It is therefore possible that hyposmia, severe hyposmia, and anosmia directly relate to SARS-CoV-2 infection severity. Further research is needed to confirm these findings in a larger cohort of CoViD-19 patients and to better understand how SARS-CoV-2 impacts the olfactory pathway. Finally, the smell test assessment could be a potential screening that might contribute to the decision to test suspected cases or guide quarantine instructions.

## Data Availability Statement

The raw data supporting the conclusions of this article will be made available by the authors, without undue reservation.

## Ethics Statement

The studies involving human participants were reviewed and approved by Comitato Etico delle Province di Chieti e Pescara e dell'Universita' degli Studi G. d'Annunzio di Chieti-Pescara Segreteria Tecnico-amministrativa Via dei Vestini 29B - 66100 Chieti http://comitatoetico.unich.it/. The patients/participants provided their written informed consent to participate in this study.

## Author Contributions

AM elaborated the data, wrote the manuscript, and ideated the work with GN who coordinated it. GN, NO, VP, and CD revised the manuscript. SM, EP, FC, JV, and DD'A tested the CoViD-19 patients and provided all clinical information. NO and CF tested the positive control. CD tested the negative control. All authors contributed to the article and approved the submitted version.

## Conflict of Interest

AM is the O.S.T. test inventor. The remaining authors declare that the research was conducted in the absence of any commercial or financial relationships that could be construed as a potential conflict of interest.

## Supplementary Material

Supplementary Figure 1 The O.S.T. test used in the study. **(A)** The kit box including four vials (white, the control; green, yellow, and red, exponential growing of butanol concentration), and the app used for testing is shown on a mobile phone. **(B)** Test guide and **(C)** app screen shot example showing the possible choice NO or YES (in Italian is SI).
