# Supplementary figures and images for: Smell and Taste in Severe CoViD-19: Self-Reported vs. Testing
Source: Front Med (Lausanne). 2020 Dec 2;7:589409. doi: 10.3389/fmed.2020.589409 (PMC7745760; doi:10.3389/fmed.2020.589409)

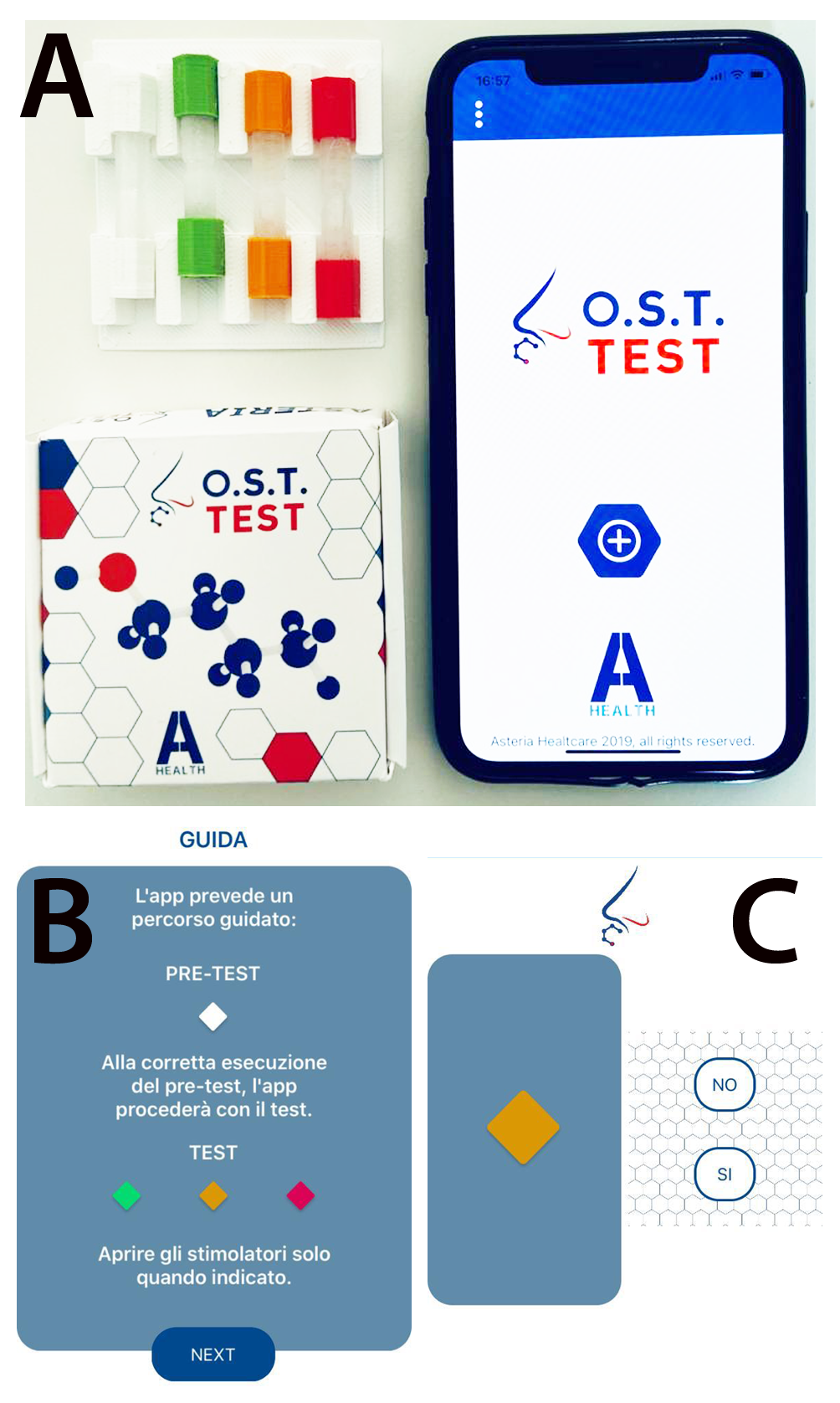

Supplement: Supplementary Figure 1 — The O.S.T. test used in the study. (A) The kit box including four vials (white, the control; green, yellow, and red, exponential growing of butanol concentration), and the app used for testing is shown on a mobile phone. (B) Test guide and (C) app screen shot example showing the possible choice NO or YES (in Italian is SI). [file Image_1.TIF]
